# Gastrojejunal Anastomotic Technique. Does It Matter? Weight Loss and Weight Regain 5 Years After Laparoscopic Roux-en-Y Gastric Bypass

**DOI:** 10.1007/s11695-020-04932-3

**Published:** 2020-08-26

**Authors:** Matyas Fehervari, Khaled Alyaqout, Ali Lairy, Haris Khwaja, Gianluca Bonanomi, Evangelos Efthimiou

**Affiliations:** 1 Department of Bariatric and Metabolic Surgery, Chelsea and Westminster Hospital, London, UK; 2 Imperial College London, London, UK; 3 Jaber Alahmad Alsabah Hospital, Kuwait City, Kuwait; 4 Mubarak Al Kabeer Hospital, Jabriya, Kuwait

**Keywords:** Gastric bypass, Weight loss, Gastrojejunal anastomosis, Anastomotic technique, Gastrojejunal stoma size, Weight regain, Surgical technique, Anastomosis size, Laparoscopic roux-en-Y gastric bypass, LRYGB

## Abstract

**Purpose:**

The gastrojejunostomy during laparoscopic Roux-en-Y gastric bypass (LRYGB) can be constructed by hand sewn (HSA), linear (LSA) and circular (CSA) stapler technique. They are all considered safe; however, it is not known which the best technique is. Short-term follow-up suggest no difference in weight loss or weight regain between them. However, there is no information on these parameters in the long term. Theatre time and cost are other important factors defining the best way to form gastrojejunostomy.

**Materials and Methods:**

In a prospective longitudinal cohort study consecutive patients following primary LRYGB were recruited to a bariatric database in a tertiary care centre. Anastomotic technique, diameter, the length of operations and associated costs, weight loss and weight regain were recorded. Patients were followed up for 5 years.

**Results:**

A total of 385 patients with an initial body mass index of 47.1 kg/m^2^ (35–68) were enrolled to this study. This decreased to 33.3 kg/m^2^ (21–54 kg/m^2^) after 5 years. There was no difference in %TWL after 3 years, *P* = 0.296, or 5 years, *P* = 0.187, between the techniques. The number of patients with weight regain was not different after 3 years, *P* = 0.224, or 5 years, *P* = 0.795. All techniques had similar operative time. CSA has a higher material cost. Early anastomotic stricture was more common following HSA; however, the difference was not significant.

**Conclusion:**

Mid-term weight loss and weight regain are not related to anastomotic technique, and there is no difference in operative time associated to them. Circular stapler technique has a higher material cost due to the additional stapler.

## Purpose

Obesity is a chronic disease affecting 13% of the adult population in western societies [1, 2]. The increased prevalence of obesity led to the evolution of metabolic surgery, which is currently the only effective way of improving medical comorbidities and achieving long-term weight loss [3–5]. Gastric bypass as a bariatric procedure was introduced by Mason et al. in 1967 [6] and remains the gold standard bariatric procedure now performed via laparoscopic approach (LRYGB) [7, 8].The evolution of this procedure aimed at improving weight loss and co-morbidities with the least complications led to multiple variations including different lengths of bilio-pancreatic, alimentary limb and common channel [9, 10], difference in gastric pouch size and shape [11], or a variety of anastomotic techniques.

The three most common surgical techniques forming the gastrojejunostomy are hand-sewn (HSA), linear stapler (LSA) and circular stapler (CSA) anastomosis [12]. These techniques are often used based on a surgeon’s preference determined by their training and experience. So far, they have only been extensively compared from a safety point of view. Previous studies were mostly focusing on prevalence of anastomotic leaks and other complications such as early stricture, infection or bleeding [13–15]. There are pros and cons for each technique, but currently, they are all considered safe and are accepted to form gastrojejunostomy. Less evidence is available on how different anastomotic techniques can influence weight loss and weight re-gain, particularly in the longer term [11]. As safety features have been now thoroughly investigated the next important step is to focus on weight loss, weight regain and other health benefits that may be associated with some of these techniques.

The available short-term weight loss results for up to 2 years suggest similar results with each technique; however, most of these studies are comparing just two techniques [16, 17]. There are even fewer studies investigating the three techniques simultaneously, with those suggesting no difference between them on the short term [18].There are no studies investigating weight loss in relation to all three gastrojejunostomy technique beyond 2 years.

The sustainability of the nadir weight is a major challenge after bariatric surgery. Approximately 10% of patient’s re-gain significant weight 3 years after LRYGB and 30% on longer term [19, 20]. These individuals’ insulin resistance, diabetes, depression and other comorbidities re-occur, and their Quality of Life decreases [21, 22]. Changes to the structure and diameter of the gastroenterostomy are described as a major factor in weight re-gain, which is influenced by surgical technique. However, weight regain following these gastrojejunostomy techniques has not been evaluated over midterm or long term [23, 24].

Operative time and cost are another important issue which has been inconsistently reported. There are suggestions that stapled mechanical anastomoses have advantages in terms of operative time; however, other studies were not able to demonstrate a difference [15, 25].

This is the first study comparing three different gastrojejunal anastomotic techniques in terms of weight loss over a 5-year period of time. A cost analysis and average operative time in a tertiary care NHS centre have also been evaluated.

## Materials and Methods

In a longitudinal cohort study, consecutive patients undergoing primary LRYGB using three different techniques were recruited at the Department of Bariatric and Metabolic Surgery, Chelsea and Westminster Hospital, London, UK, a tertiary care centre. Patients with a BMI higher than 35 kg/m [2] were prospectively registered into a local database between 1 October 2009 and 31 March 2015. Patients were followed up for 5 years. Only patients who had LRYGB as a primary weight loss procedure were included. All patients completed a bariatric pathway and were cleared by a multidisciplinary team. The medical history and physical examination findings were recorded at initial presentation and subsequent follow-ups. Relevant demographic and general clinical data were also recorded. Three bariatric surgeons operated on all cases with each surgeon using one of the three techniques; surgeons were allocated by the MDT with no consideration of source of referral or patient related factors such as initial weight or comorbidities.

### Surgical Technique and Perioperative Care

All patients underwent a comprehensive pre-operative assessment. According to current guideline patients were starved for a standard 6 h to solids and 2 h to liquids before their operation [26]. Patients were weighed on the morning of surgery. Three different gastrojejunal anastomotic techniques were performed. These were hand sewn, linear or circular stapled anastomoses. The exact surgical techniques used in our Institution were described previously [18]. These techniques were standardized in order to reproduce the exact same anastomosis in each case. The diameter of the anastomoses was also standardized for each technique respectively HSA 11–12 mm, LSA 20 mm and CSA 25 mm. The biliary limb was 60 cm, and the alimentary limb was 100 cm with all techniques.

All bypasses were constructed in an antecolic antegastric fashion. Routine methylene-blue dye test was performed intraoperatively and postoperatively. Intraoperative leak test was performed by administering 100–150 ml of methylene blue through an orogastric tube whilst simultaneously obstructing the alimentary limb close to the anastomosis. Once the gastric pouch visibly distended the area of the anastomosis was inspected for methylene blue. A drain was placed immediately posterior to the staple line. Postoperative methylene blue leak test was performed by asking the patient to drink 200 ml of methylene blue at 7:00 am on the first postoperative morning. Five hours later the drain output was inspected for methylene blue by a senior Doctor. Once anastomotic leak was ruled out patients were commenced on fluids on day one and progressed on to a pureed diet on the following days. Patients received the same post-operative care including dietary advice. Follow-up of patients beside the Surgical Team were carried out by specialist nurses, allied healthcare professionals, and other medical teams such as endocrinology/obesity medicine. The average cost of theatre time in the NHS was calculated in existing health data and suggested to be £15/min [27].The cost of consumables were calculated based on each Consultant Procedure Card by registering the cost of each item as recorded in the hospitals’ inventory and adding them together.

### Assessment of Weight Loss and Weight Regain

Percentage of total weight loss (TWL%), percentage excess weight loss (EWL%) and BMI were calculated as previously described by Brethauer et al. [28]. Nadir weight was determined as the lowest weight during the first 3 or 5 years of the follow-up [29]. As described by Lauti et al. and Baig et al. weight regain (WR) was defined by either regaining more than 25% of weight lost from the NADIR weight or gained more than 5 BMI points from the BMI calculated at NADIR weight [23, 30].

### Stricture

Strictures were evaluated over the first 90 days following the operation. Patient with symptoms suggestive of a stricture (dysphagia, inability to progress with diet, nausea, vomiting, epigastric pain resistant to PPI) were evaluated with UGI endoscopy. Gastrojejunal anastomotic strictures were diagnosed if it was not possible to insert a standard 10-mm gastroscope through the anastomosis [18].

### Statistical Analysis

Statistical analysis was performed with Prism for Windows 5.01 (GraphPad Software, San Diego, CA) and SPSS for Mac OSX 21.0.0 (SPSS Inc., Chicago, IL) statistical software products. As many of the variables had non-Gaussian distributions, we used nonparametric tests for the analysis. We used the Mann–Whitney’s *U* test to compare two independent groups and Kruskal–Wallis one-way analysis of variance (ANOVA) for comparing multiple groups. Contingency tables were analysed by Fisher exact test and chi-square test. Statistical analyses were performed two-tailed, and *P* < 0.05 was considered as significant. Values presented in the text are median, minimum and maximum values in brackets unless otherwise stated. The study protocol was approved by Research and Development Office at our Institution (Reference number: PCD884).

## Results

### Clinical Characteristics

Exactly 385 patients were recruited prospectively to our database. The majority of patients (315) were female; the median age was 46 (19–67) years. At the time of the presentation to the Bariatric Programme there were 54 (14%) current and 93 (24%) ex-smokers. All smokers had to give up smoking for a period of 6 months prior to surgery. Exactly 123 (31.9%) patients had diabetes, 149 (38.7%) suffered with hypertension, 70 (18.1%) had sleep apnoea and 144 (37.4%) were diagnosed with GORD. Exactly 370 (96.1%) patients were followed up for 1 year, 346 (89.8%) for 3 years and 311(80.7%) for 5 years. There was no difference in baseline clinical characteristics between patients with different follow-up times.

LRYGB with hand sewn anastomosis was performed in 198 cases. Linear staplers were used for 132 patients and circular staplers in 55 cases. Following 1 year 192 were followed with HSA, 124 with LSA and 54 with CSA. Exactly 176 with HSA, 119 with LSA and 51 patients with CSA were followed up for 3 year. A total number of 311 patients were followed up for 5 years. Out of these 155 patients underwent HSA, 108 LSA and 48 CSA.

### Weight Loss

The overall initial BMI was 47.1 kg/m^2^ (35–68), and the weight was 129.1 kg (79–209). Following 1 year the BMI decreased to 31.9 kg/m^2^ (19–54); overall weight was 89 kg (51–155), which correspond to a BMI change of − 14.7 kg/m^2^ [3–30]. The overall %TWL was 31.0% (5–55), and %EWL was 66.3% (9–140) 1 year after Roux-en-Y gastric bypass. After 3 years the BMI was 32.4 kg/m^2^ (20–53), which correspond to a median weight of 88 kg (50–149). The mean BMI decrease was 14.4 kg/m^2^ (2–34). The overall %TWL was 30.6% (4–55), and %EWL was 65.9% (9–126) 3 years following bariatric surgery. At the end of the 5 years follow-up period the BMI was 33.3 kg/m^2^ (21–54) and weight was 91.2 kg (56–155). This represents an overall decrease of 13.6 kg/m^2^ (0–36). The %TWL was 28.8% (0–58), and the %EWL was 62.04% (0–122).

**Fig. 1:**
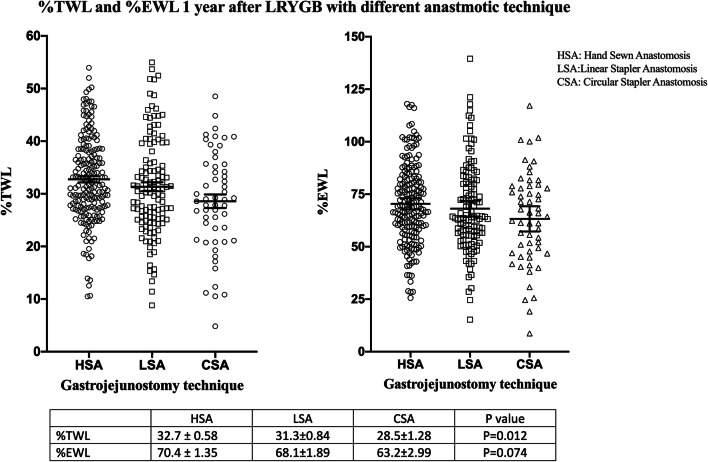
Scatterplot diagram of percentage total and excess weight loss 1 year after surgery. Kruskal–Wallis one-way ANOVA, values are mean ± standard error of mean

**Fig. 2:**
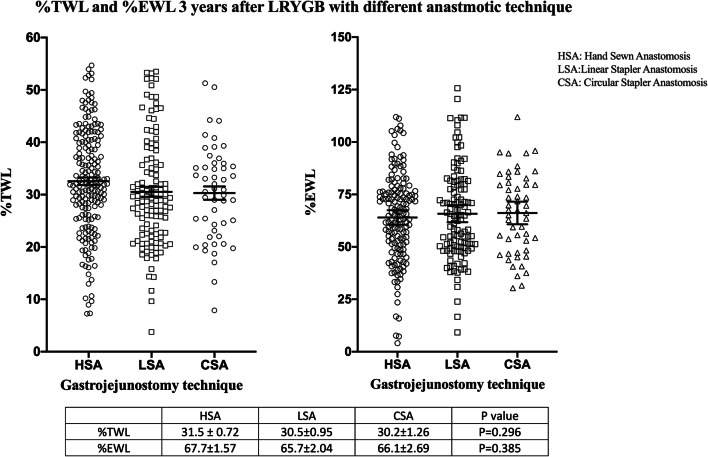
Scatterplot diagram of percentage total and excess weight loss 3 years after surgery. Kruskal–Wallis one-way ANOVA, values are mean ± standard error of mean

**Fig. 3:**
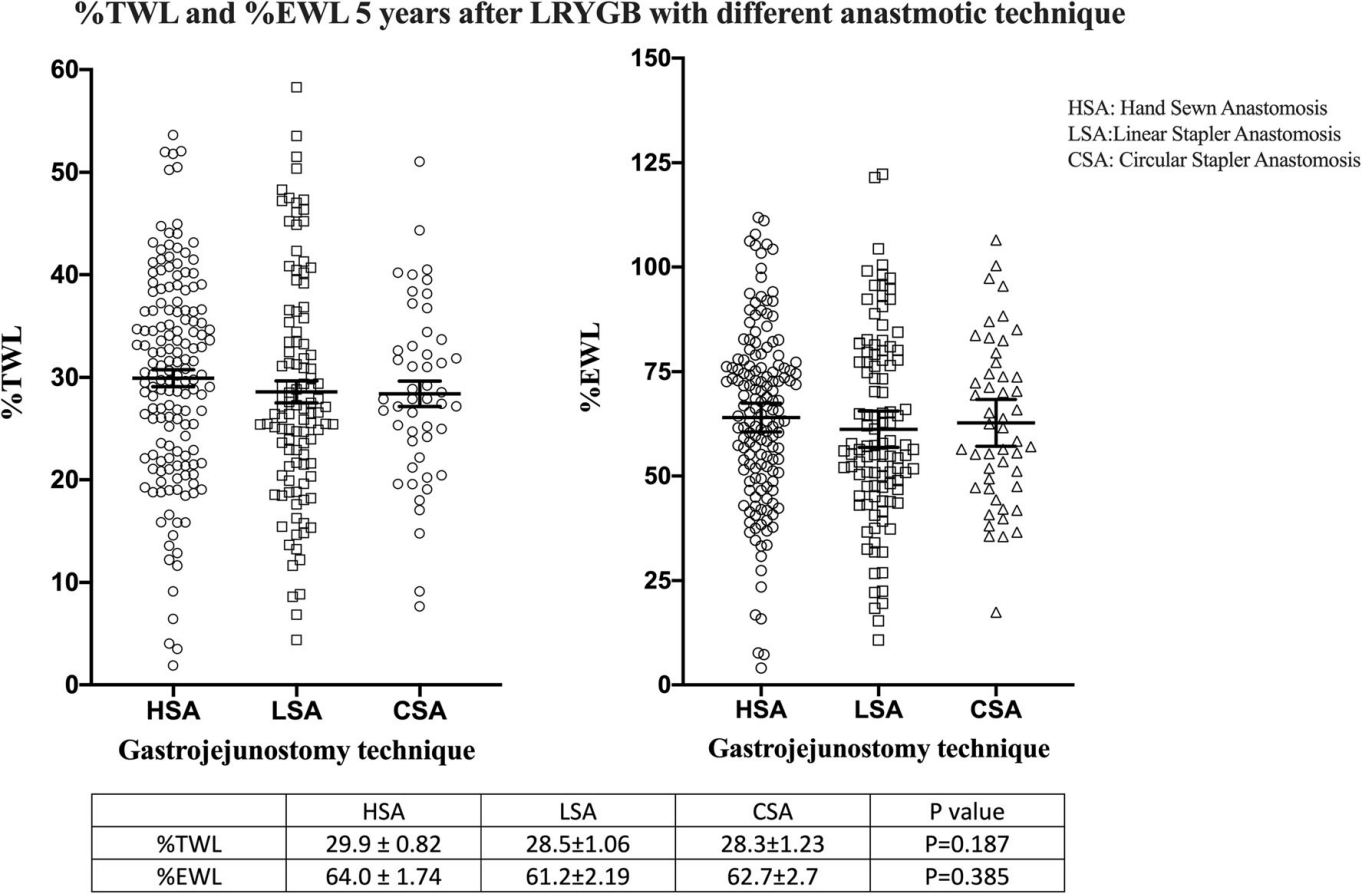
Scatterplot diagram of percentage total and excess weight loss 5 years after surgery. Kruskal–Wallis one-way ANOVA, values are mean ± standard error of mean

The three groups were compared with Kruskal–Wallis one-way ANOVA. Results following 1 year demonstrated that there was a difference in %TWL, *P* = 0.012, and there was no difference in %EWL, *P* = 0.074, between the groups (Fig. 1). As displayed in Fig. 2, there was no difference between the groups in %TWL, *P* = 0.296, or %EWL, *P* = 0.385, after 3 years. Following 5 years the %TWL and %EWL were equal between the groups respectively *P* = 0.187 and *P* = 0.385 (Fig. 3.).

Further comparison has been conducted between two different groups with Mann–Whitney *U* test. After 1 year there was no statistically significant difference in %TWL between HSA and LSA *P* = 0.061 and LSA and CSA *P* = 0.185. The %EWL was similar between HSA and LSA *P* = 0.141 and LSA and CSA *P* = 0.295. There was a difference between HSA and CSA both %TWL, *P* = 0.006, and %EWL, *P* = 0.039. After 3 years there was no difference in TWL (%), between HSA and LSA, *P* = 0.129; LSA and CSA, *P* = 0.776; and HSA and CSA, *P* = 0.419. There was also no statistically significant difference in %EWL between any two groups: HSA and LSA, *P* = 0.262; LSA and CSA, *P* = 0.753; and HSA and CSA, *P* = 0.684. Comparison of two groups with MWU test 5 years after LRYGB suggested no difference in %TWL respectively, HSA and LSA, *P* = 0.101; LSA and CSA, *P* = 0.725; and HSA and CSA, *P* = 0.205. Evaluating the %EWL after 5 years similarly suggested no difference between HSA and LSA, *P* = 0.183; LSA and CSA, *P* = 0.621; and HSA and CSA, *P* = 0.501.

### Weight Regain and Stricture Rate

The incidence and comparison of weight regain displayed in Table 1. Out of the 32 patients regaining weight during the first 3 years without surgical intervention only 3 (9%) were able to reduce this below 125% of their nadir weight by the end of the 5 years follow-up (one of each technique). The different techniques were compared with each other with Fisher’s exact test. There were no significant difference between HSA and LSA (*P* = 0.102), LSA and CSA (*P* = 0.331) or HSA and CSA (*P* = 1) after 3 years. At the end of the follow-up period there were no difference between the three techniques in weight regain, respectively HSA and LSA (*P* = 0.892), LSA and CSA (*P* = 0.577) and HSA and CSA (*P* = 0.600).

**Table 1:** Number and percentage of patients with weight regain and early anastomotic stricture

	Hand sewn	Linear stapler	Circular stapler	*P* value (Kruskal–Wallis ANOVA)	Total
WR at 3 years	20/173 (11%)	7/123 (6%)	5/50 (10%)	*P* = 0.224	32 (9.2%)
WR at 5 years	48/153 (31%)	33/110 (30%)	17/48 (35%)	*P* = 0.795	98 (31.5%)
Stricture by 90 days	12(6.3%)	7 (5.1%)	2 (3.6%)	N/A	21 (5.45%)

The incidence of anastomotic stricture requiring endoscopic dilatation across the different groups was evaluated over the first 90 post-operative days. Results are displayed in Table 1. Patients developing anastomotic strictures lost significantly more weight than patients without stricture %TWL (*P* = 0.038) and %EWL (*P* = 0.040).

### Cost of the Different Techniques

The median theatre time for HSA was 140 min (69–249 min); for CSA it was 138 min (101–240 min), and for LSA 140 min (65–231 min).

Comparing the three groups with Kruskal–Wallis ANOVA no significant difference was found amongst them (*P* = 0.892).

The material cost of the different anastomotic technique was collected as described in our methods and combined with the cost of theatre time (Table 2.). All surgeons and medical staff were on the same pay scale determined centrally by the government***.***

**Table 2:** Estimated costs of different anastomotic techniques

	HS	CS	LS
Theatre time cost	£2100	£2070	£2100
Material cost	£1929	£2736	£1912
Overall cost	£4029	£4806	£4012

## Conclusions

This is the first study comparing weight loss and the incidence of weight re-gain over 5 years in relation to the three most common gastrojejunal anastomotic techniques. Our results are similar to short-term results suggesting that midterm weight loss is equivalent between the three techniques.

The significant co-morbidities of our cohort represents a morbidly obese population with significant past medical history. HSA was performed most frequently followed by LSA and CSA over the study period. The percentage of patient followed up in the present study is statistically appropriate, demonstrative and comparable to previous studies [31].Weight loss is defined by multiple standard measures as previously suggested [28]. The degree of overall weight loss and the proportion of patients suffering weight regain after LRYGB are comparable to data recorded in large volume multicentre studies [20].

The most important finding of this study is that weight loss and weight regain are the same 3 and 5 years after LRYGB regardless of anastomotic technique. This suggests that in midterm there is no superior GJA anastomotic technique that results in better weight loss. The three types of anastomosis have different diameters. HSA is the narrowest by approximately half of the diameter of CSA and LSA. Initial anastomotic diameter was found important within CSA technique in developing early strictures [32]. Our results suggest that diameter has less importance when comparing HSA to LSA. Despite the two-fold difference of the anastomotic diameter, the stricture rate and the amount of weight loss are both similar. Proportionally, there are more strictures following HSA and LSA than following CSA. Previously, it was suggested that LSA may be followed by less strictures than CSA, which we cannot confirm in this study [14]. Patients developing a stricture lost significantly more weight than the rest of the patients, which explains why HSA has a higher one-year %TWL and %EWL than CSA.

Treatment and prevention of weight regain is one of the current challenges of metabolic surgery. There are several factors associated with weight regain such as anastomotic dilatation and pouch expansion. Different surgical techniques may alter the way anastomosis evolve by time; hence, it was crucial to assess the incidence of weight regain following each technique. Three years after the operation there was no difference between the numbers of patients suffering weight regain in the different groups. Only 3 (9%) of the patients who regained significant amount of weight were able to lose it without any intervention by 5 years. In accordance with previous studies, approximately 30% of patients regained weight in our series by the end of the follow-up period. The distribution of patients suffering weight regain is the same across the three groups, which suggests that anastomotic technique has no effect on the mid-term weight regain.

Theatre time is a major contributor to the overall cost of procedures. There is a lot of controversiality around the length of operative time in the literature most likely due to multiple factors such as operative set up, time for training and differences in effective team work. The most robust studies suggest little difference between them. This is confirmed in our study as well demonstrating no significant difference in the length of the operations with different techniques. This suggests that the length of operations is more likely to be operator rather than technique dependent. Every surgeon is likely to perform better with the technique they are more experienced and comfortable with. The overall cost analysis in our tertiary centre in the UK suggests that LSA and HSA have similar costs, while the CSA technique entails the additional price of the circular stapler and Orvil device.

The most important finding of this study was to demonstrate that equal amount of sustainable weight loss can be achieved with either of these techniques. In addition, theatre time is more likely to be surgeon dependent. The cost of equipment is higher with CSA, whilst early strictures are relatively more common after HSA.

In conclusion, we were not able to demonstrate a superior gastrojejunostomy technique over 5 years following LRYGB. However, more research, randomized multicentre studies and evaluation of remission and reappearance of comorbidities secondary to weight regain are needed before drawing final conclusion on which is the best anastomotic technique.
